# Genomic surveillance of SARS-CoV-2 in Weihai, China, march 2022 to march 2023

**DOI:** 10.3389/fpubh.2023.1273443

**Published:** 2023-11-14

**Authors:** Xiang Li, Yuwei Zhang, Jinbo Zhang, Zongyan Sui, Xinyi Qu, Mingrui Wang, Tingting Miao, Jizhao Li

**Affiliations:** ^1^Weihai Center for Disease Control and Prevention, Weihai, China; ^2^Shandong Center for Disease Control and Prevention, Jinan, China

**Keywords:** COVID-19, SARS-CoV-2, genomic surveillance, Weihai, China

## Abstract

COVID-19 is an acute respiratory infectious disease caused by SARS-CoV-2. It was first reported in Wuhan, China in December 2019 and rapidly spread globally in early 2020, triggering a global pandemic. In December 2022, China adjusted the dynamic COVID-zero strategy that lasted for three years. The number of positive cases in China increased rapidly in the short term. Weihai was also affected during this period. We conducted genomic surveillance of SARS-CoV-2 variants in Weihai during this period, hoping to understand the changes in the genomic characteristics of SARS-CoV-2 before and after the adjustment of the epidemic policy. In this study,we collected SARS-CoV-2 positive samples from March 2022 to March 2023 in Weihai and performed SARS-CoV-2 whole genome sequencing on these samples using next-generation sequencing technology. we obtained a total of 704 SARS-CoV-2 whole genome sequences, and selected 581 high-quality sequences for further analysis. The analysis results showed that from March 2022 to November 2022, before the adjustment of epidemic policy, the COVID-19 cases in Weihai were mainly from four local clusters,which were caused by four variants, including BA.2,BA.1.1,P.1.15 and BA.5.2.1. Phylogenetic analysis showed that: In the same cluster,the sequences between each other were highly homologous, and the whole genome sequence were almost identical. After December 2022, the epidemic policy was adjusted, BF.7 and BA.5.2 became the dominant variants in Weihai, consistent with the main domestic strains in China during the same period. Phylodynamic analysis showed that BF.7 and BA.5.2 had a large amount of genetic diversities in December, and the effective population size of BF.7 and BA.5.2 also showed explosive growth in December. In conclusion, we reported the composition and dynamic trend of SARS-CoV-2 variants in Weihai from March 2022 to March 2023. We found that there have been significant changes in the variants and expansion patterns of SARS-CoV-2 before and after the adjustment of epidemic policies. But the dominant variants in Weihai were the same as the SARS-CoV-2 variants circulating globally at the same time and we found no persistently dominant variants or new lineages during this period.

## 1. Introduction

COVID-19 is an acute respiratory infectious disease caused by SARS-CoV-2. It was first reported in Wuhan, China, in December 2019 (1) and then rapidly spread to worldwide, causing at least 6.9 million deaths in the following 3 years (2).

SARS-CoV-2 belongs to the family *Coronavirus*, genus *Betacoronavirus* (3). It can bind to the ACE2 receptor on the cell surface and invade the human body through the upper respiratory tract (4). With widespread transmission and continuous evolution,SARS-CoV-2 has generated numerous variants (5–7). The WHO classified these SARS-CoV-2 variants that require focused attention into variants of concern (VOC). Currently, there are five VOCs, Alpha, Beta, Gamma, Delta and Omicron (8). Omicron was first detected in South Africa in December 2021 (9). With its strong transmissibility, it quickly replaced other variants and became the current dominant variant.

Weihai is a coastal city in northern China with one international airport and three major ports. Since the outbreak of the COVID-19 in Wuhan in 2020, China began to adopted dynamic COVID-zero strategy (10). Weihai also complied with these policies during this period. After December 2022, with the pathogenicity of the Omicron variant weakened (11–13), China adjusted its epidemic prevention policies and the number of positive cases in China increased rapidly in the short term (14). The changes of SARS-CoV-2 variants circulating in China during this period have also received widespread attention.

In this study, we collected positive samples of SARS-CoV-2 from March 2022 to March 2023 in Weihai, which are consists of local cases and foreign imported cases. We performed whole genome sequencing and conducted variation analysis, phylogenetic analysis on these samples. Through the analysis of these data, we hope to understand the changes in the genome characteristics of SARS-CoV-2 in Weihai before and after the adjustment of epidemic policies.

## 2. Materials and methods

### 2.1. Sample collection

SARS-CoV-2 nucleic acid positive samples were collected in Weihai from March 2022 to March 2023. These samples consisted of throat swab samples from local cases and foreign imported cases. Samples of local cases were provided by hospital clinical laboratories, district Centers for Disease Control Prevention and Control (CDC) and third-party testing laboratories.Samples of foreign imported cases were provided by Weihai Customs Laboratory. These samples were tested positive by using Real-time PCR before we received them. We retested these samples and selected positive samples with CT < 32 for whole genome sequencing.

### 2.2. Whole-genome sequencing

The viral RNA was extracted by Automatic nucleic acid extraction instrument (Jiangsu Bioperfectus Technologies Co,Ltd). Using the viral RNA as a template, the whole genome of SARS-CoV-2 was amplified by PCR using the 2019-nCoV Genome Capture Kit (MicroFuture, Beijing, China). The PCR products were purified using AMPure XP magnetic beads. Library construction was performed using the Nextera XT DNA Library Preparation Kit and Nextera XT Index Kit (Illumina, San Diego, CA, United States). Whole genome sequencing was performed using Illumina Nextseq2000 sequencer and NextSeq 1000/2000 P1 reagents (300 cycles) (MS-102-2002).The sequencing read length was set as 2 × 151.

### 2.3. Variant analyses and phylogenetic analyses

SARS-CoV-2 genome aligment and assembly were performed using CLC Genomics Workbench version22.0. Wuhan-Hu-1 (NC_045512.2) was used as the reference sequence, and QC analysis, trim and map were performed on the FASTQ file to obtain the spliced SARS-CoV-2 whole genome sequence. Nextclade (15) (https://clades.nextstrain.org/ Version2.13.0) was used to perform clade typing, pangolin typing, QC, and mutation site analysis on the obtained SARS-CoV-2 whole genome sequences. Based on the typing results, the relevant sequences were downloaded from the GISAID database, and the phylogenetic tree was constructed using MEGA version11.The Neighbor-Joining (NJ) method was used to construct the evolutionary tree and the Bootstrap value was set to 1,000 times to evaluate the reliability of the phylogenetic tree.

BF.7 and BA.5.2 sequences collected from Weihai after December 2022 was performed phylodynamic analysis.Firstly, PhyloSuite (16) version v1.2.212 was used to match the best substitution model. Based on the analysis results, the best substitution model was TIM with 4 Gamma category count.Then TempEst (17) version 1.5.313 was used to detect whether there was enough temporal molecular signal for phylodynamic analysis. The analysis results indicated that after removing some outliers, there was enough temporal molecular signal for subsequent analysis.BEATUti was used for parameter setting, set the clock model to strict molecular clock model, set the tree priors to coalescent Bayesian skyline, and set the chain length to 100million (18). We used BEAST version 2.7.4 to run the XML file generated by BEATUti.Tracer version 1.7.2 was used to check the convergence of MCMC chains (ESS > 200) and performed Bayesian Skyline Analysis to estimate the effective population size for both BA.5.2 and BF.7. TreeAnnotator (19) was used to remove 10% of the MCMC chains as burn-in to build a maximum clade credibility tree (MCC tree). FigTree version1.4.4 was used to add time axis to the MCC tree.

## 3. Results

### 3.1. Genetic characteristics and mutations identified of SARS-Cov-2

**Figure 1:**
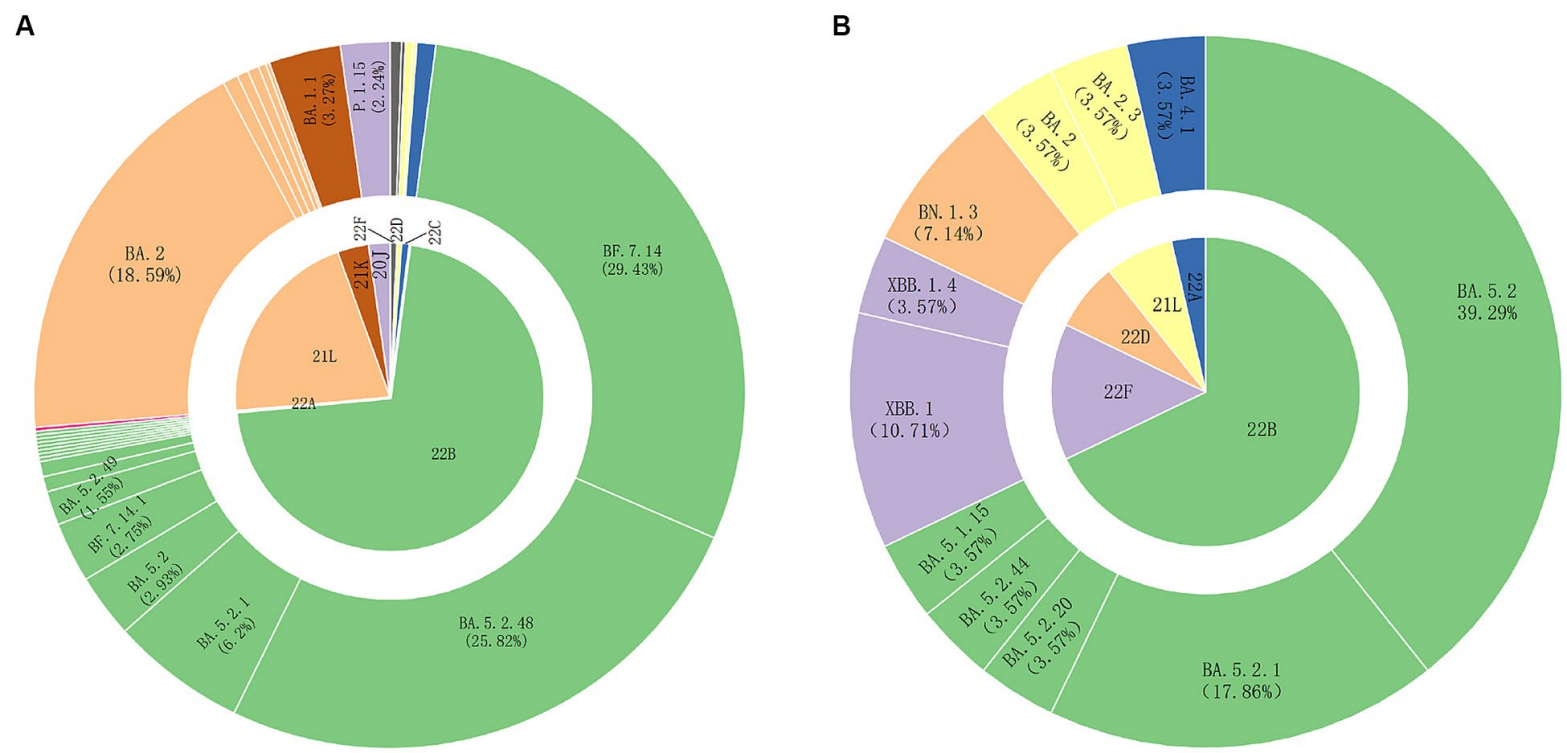
SARS-CoV-2 variant strains prevalent in Weihai from March 2022 to March 2023. **(A)** SARS-CoV-2 variant strains prevalent in Weihai from March 2022 to March 2023. The inner circle shows the results of Clade typing. The outer circle shows the Pangolin lineages. The unlabeled Pangolin lineages in the figure include XBB.1, XBB.1.4, BN.1.3, BN.1.5, BA.5.2.50, DY.1, BA.5.1.15, BA.5.1.32, BA.5.2.20, BA.5.2.44, BA.5.2.6, BF.7.14.3, DZ.1, BA.5. 2.22, BA.4.1, BA.2.76, BA.2.3.14, BA.2.2.1, BA.2.5, BA.2.3. **(B)** The foreign imported cases of SARS-CoV-2 variant strains prevalent in Weihai from March 2022 to March 2023. The inner circle shows the results of Clade typing. The outer circle shows the Pangolin lineages.

We collected SARS-CoV-2 nucleic acid positive samples from March 2022 to March 2023 in Weihai, and selected samples with CT < 32 for whole-genome sequencing. We obtained a total of 704 SARS-CoV-2 whole-genome sequences. After removing some duplicate sequences and low-quality sequences, a total of 581 whole genome sequences were finally obtained for subsequent analysis. Among them, 553 sequences were local cases sequences and 28 were foreign imported cases sequences. The typing results showed that 568 sequences were Omicron variants and 13 sequences were Gamma variants. According to the Clade typing results, there were eight clades, including 22B (71.43%), 21 L (20.83%), 21 K (3.27%), 20 J (2.24%), 22C (0.86%), 22F (0.69%), 22D (0.52%), and 22A (0.17%). According to the Pangolin typing results, there were 30 Pango lineages, the predominant lineages were BF.7.14 (29.43%), BA.5.2.48 (25.82%), BA.2 (18.59%), and the other lineages accounting for more than 1% included BA.5.2.1 (6.20%), BA.1.1 (3.27%), BA.5.2 (2.93%), BA.7.14.1 (2.75%), P.1.15 (2.24%), and BA.5.2.49 (1.55%) (Figure 1A).

From March 2022 to March 2023, we obtained 28 SARS-CoV-2 whole genome sequences of foreign imported cases. According to the Clade typing results, there were 5 clades, including 22B, 22F, 22D, 21 L, 22A and 11 Pangolin lineages, including BA.5.2, BA.5.2.1, BA.5.2.20, BA.5.2.44, BA.5.1.15, XBB.1, XBB.1.4, BN.1.3, BA.2, BA.2.3, BA.4.1 (Figure 1B). BA.5.2 and its descendant lineages were dominant in the imported cases (64.28%).The Pangolin lineages of imported cases were generally consistent with those detected in local cases during the same period.However,some rare variants were also detected in the imported cases, such as BA.4.1, XBB.1, BA.5.1.15, BN.1.3, which did not appear in the local cases.

**Figure 2:**
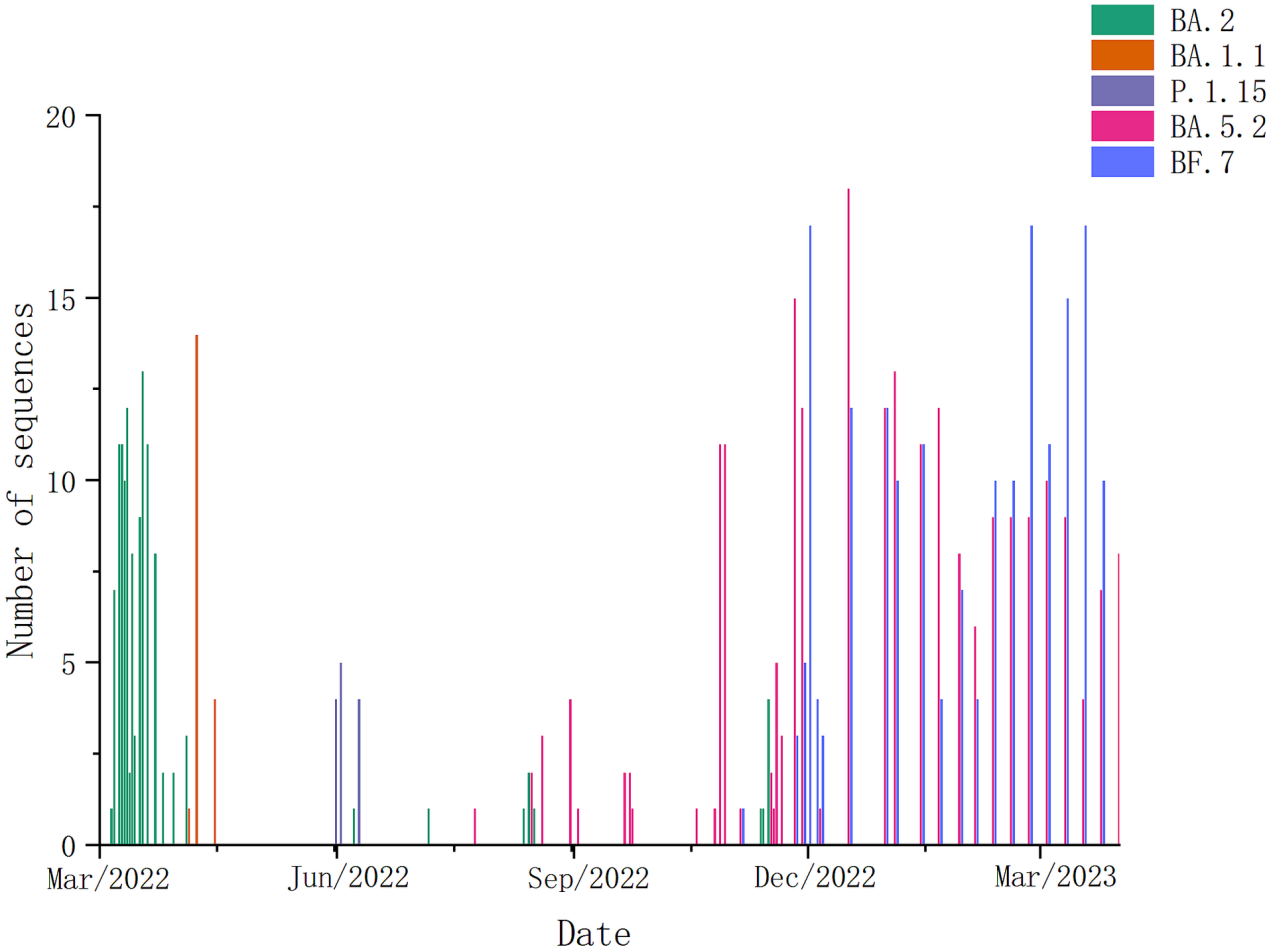
Composition and proportion of SARS-CoV-2 variants prevalent in Weihai in different months from March 2022 to March 2023. Branches with different color markings represent different Pangolin lineages.

According to the temporal distribution of SARS-CoV-2 variants in Weihai, we could find that from March to April 2022, the dominant strains in Weihai were BA. 2. From May to November 2022, the dominant strains were BA. 1.1, P.1.15, and BA. 5.2.From December 2022 to March 2023, BA.5.2 and its descendant lineages, as well as BF.7 and its descendant lineages, became the dominant strains in Weihai (Figure 2).

### 3.2. Temporal distribution of SARS-CoV-2 variants in Weihai before the adjustment of epidemic policies

**Figure 3:**
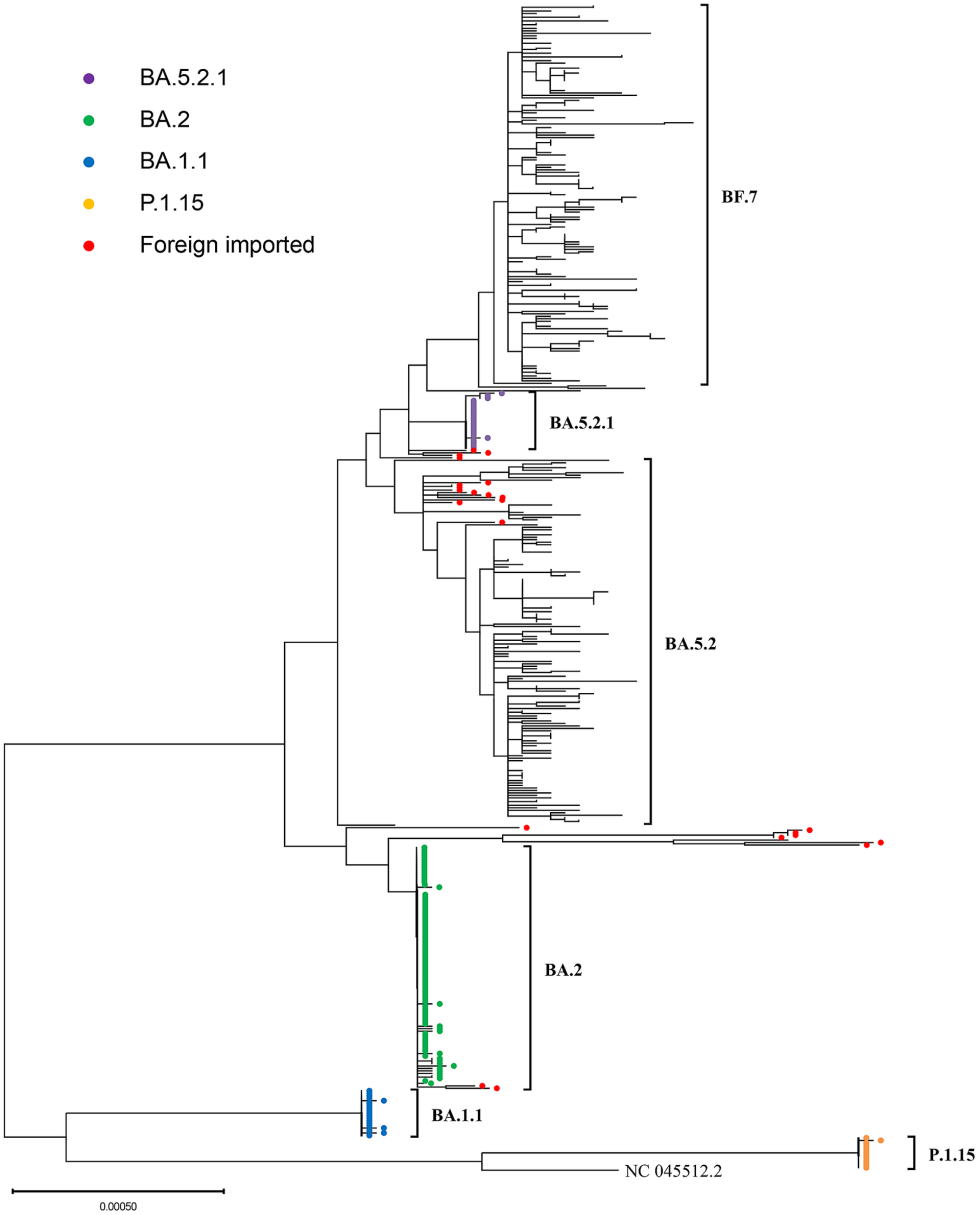
The phylogenetic tree generated from four outbreak-related SARS-CoV-2 variants in Weihai from March 2022 to March 2023. The branches marked with different color circles are the sequences of local clusters. The branches marked with red circles are the sequences of foreign imported cases.

From March 2022 to November 2022, before the adjustment of epidemic policies,we obtained a total of 277 SARS-CoV-2 whole genome sequences, including 251 local cases and 26 foreign imported cases. The variants with the highest proportion were BA.2 and its descendant lineages (45.49%). Due to the strict epidemic prevention policy, the COVID-19 cases in Weihai during this period were mainly local clusters. During this period, one large-scale local clusters and three small-scale clusters occurred in Weihai.These four clusters were caused by four different Pangolin lineages, including BA. 2, BA. 1.1, P.1.15 and BA. 5.2.1. We conducted phylogenetic analysis on the related sequences involved in these clusters, and the phylogenetic tree showed that:In the same cluster, almost all local cases sequences were on the same branch, and the gene diversities between these sequences were very limited, indicating that the sequences were highly homologous (Figure 3). The alignment results showed that their whole genome sequences were almost identical, and the SNP differences between them were no more than 3.In addition,By comparing with the imported cases in the same period.it can be found that the imported cases have the same pangolin lineages with the local cases.But they were not on the same branch in phylogenetic tree.This indicated that these four local clusters were not caused by these imported cases.By comparing with the homologous sequences downloaded from the GISAID database, we found that the local clusters of BA.2, BA.1.1 and BA.5.2.1 occurred at roughly the same time as the homologous sequences uploaded in the GISAID database. This indicated that the variants that triggered these three clusters are roughly the same as the SARS-CoV-2 variants that were circulating globally at the same time. The only exception was P.1.15, the local cluster of the P.1.15 variant occurred in June 2022, while most of the homologous GISAID database sequences were uploaded from March to July 2021.

### 3.3. Temporal distribution of SARS-CoV-2 variants in Weihai after the adjustment of epidemic policies

**Figure 4:**
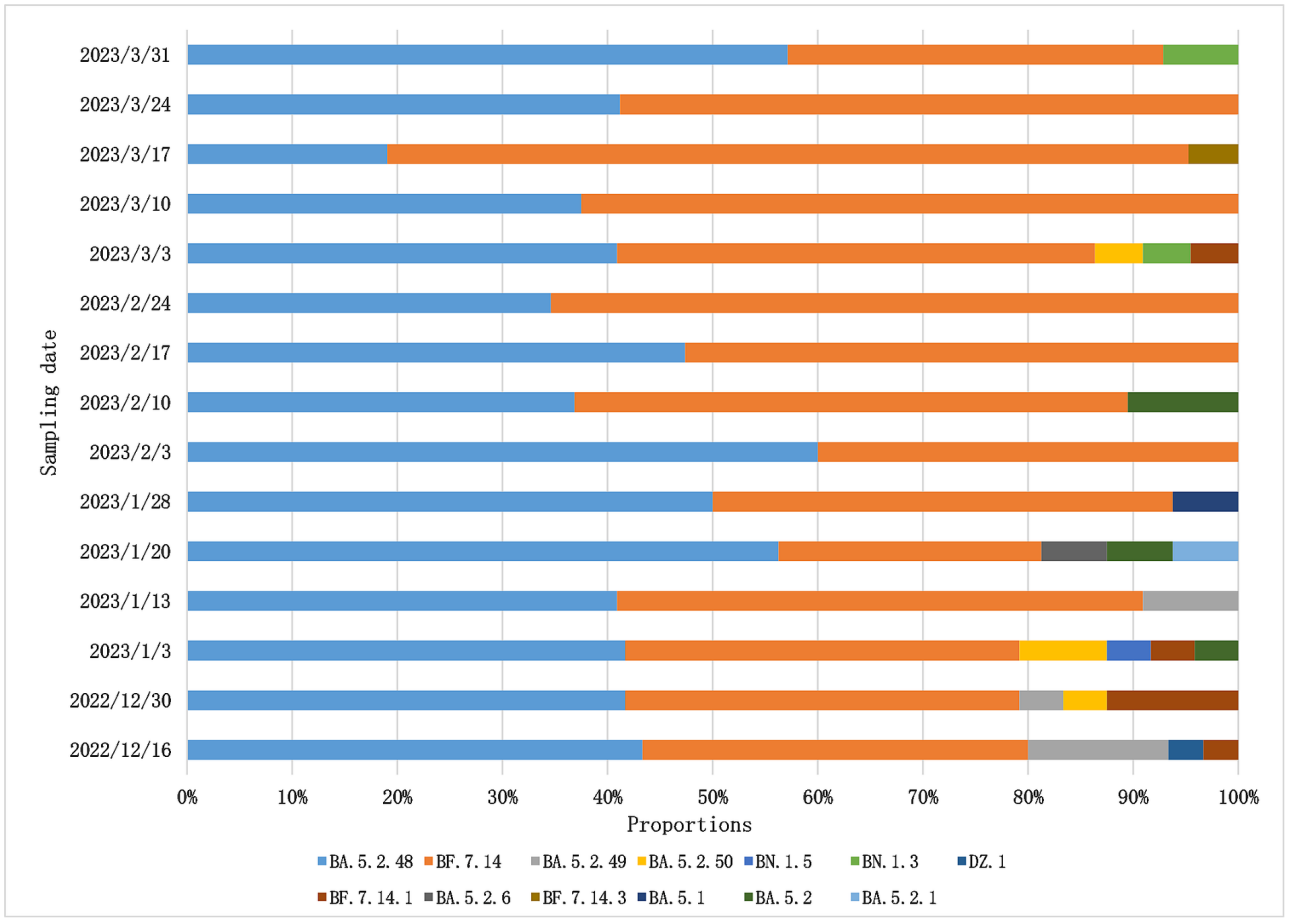
Composition and proportion of SARS-CoV-2 variants prevalent in Weihai from December 2022 to March 2023. Branches with different color markings represent different Pangolin lineages.

After December 2022, China adjusted its epidemic prevention policies and new infection cases increased rapidly in the short term,so whole-genome sequencing for SARS-Cov-2 variants was changed to once a week in Weihai. From December 2022 to March 2023, we obtained a total of 304 SARS-CoV-2 whole genome sequences.Among them, BF.7 and BA.5.2 were the two dominant variants in Weihai.BF.7 and its descendant lineages, including BF.7.14, BF.7.14.1, BF.7.14.3, BF.7.14.4,BF.7.14.5,BF.7.14.6,accounted for 50.98%. BA.5.2 and its descendant lineages, including BA.5.2, BA.5.2.1, BA.5.2.6, BA.5.2.48, BA.5.2.49, BA.5.2.50, DZ.1, accounted for 47.71%. Other variants accounted for 1.31%, including BN.1.3, BN.1.5, BA.5.1, We further analyzed the trend of the proportion of variants over time. From December 2022 to February 2023, the proportion of BA.5.2 was higher than that of BF.7. But after February, BF.7 accounted for more than BA.5.2, reaching 80.95% at the highest point (March 11–17, 2023). However, the proportion of BF.7 began to decline in late March (Figure 4). Overall, the proportions of BF.7 and BA.5.2 alternately changed over time. The proportion of BF.7 rose rapidly from February to March, but began to decline at the end of March, while the proportion of BA.5.2 declined from February to March and rebounded at the end of March.

**Figure 5:**
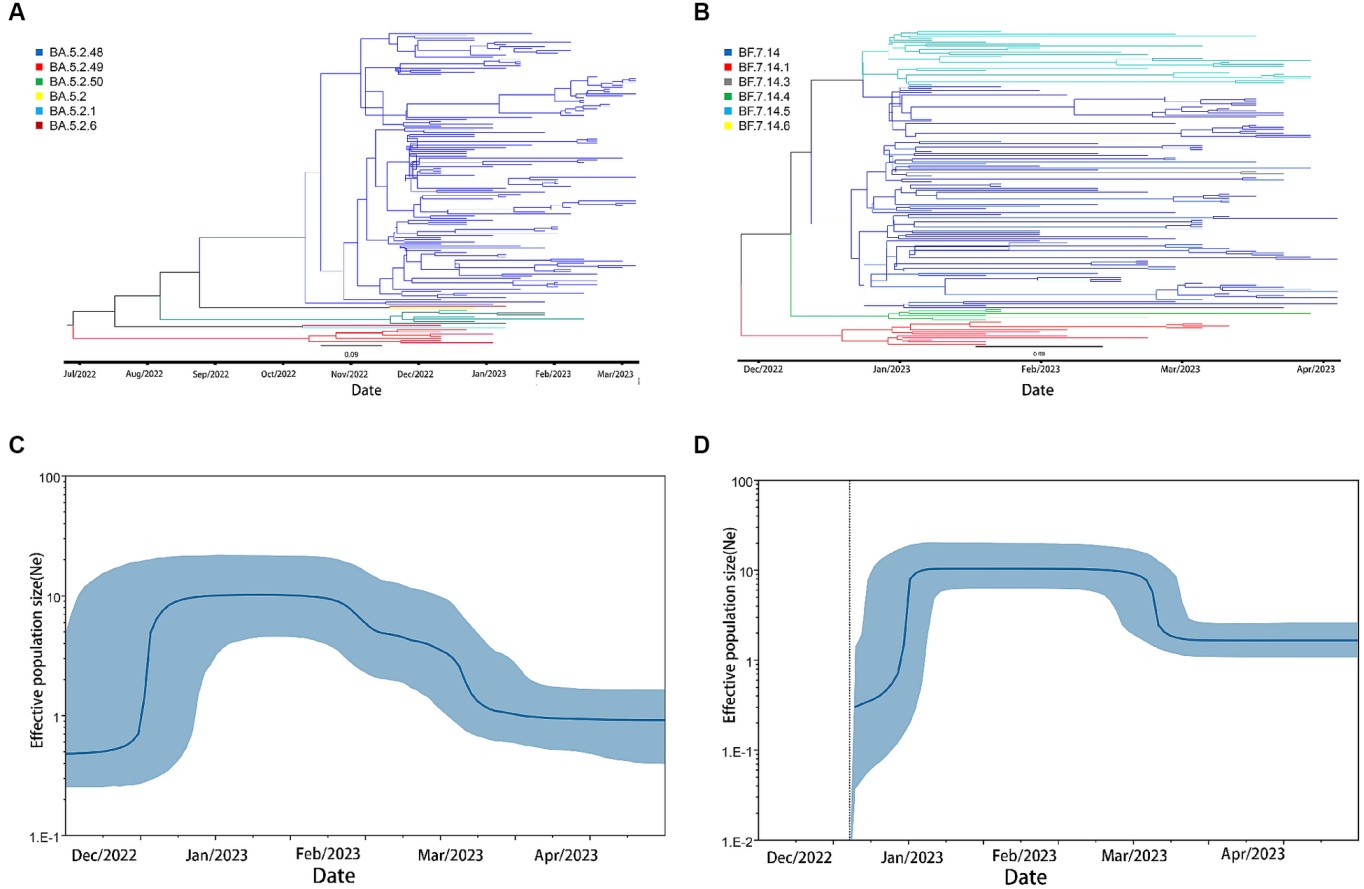
phylodynamic analysis of BA.5.2 and BF.7 in Weihai after December,2022. **(A)** The maximum clade credibility tree (MCC tree) of BA.5.2. **(B)** The Bayesian Skyline Plots of BA.5.2. **(C)** The maximum clade credibility tree(MCC tree) of BF.7. **(D)** The Bayesian Skyline Plots of BF.7.

We conducted phylodynamic analysis on the BF.7 and BA.5.2 variants from December 2022 to March 2023.The maximum clade credit tree (MCC tree) results showed that both BF.7 and BA.5.2 exhibited a large amount of genetic diversities in December 2022.The results of Bayesian Skyline Plots showed that the effective population size of BF.7 and BA.5.2 both increased exponentially during this period. BA.5.2 increased sharply around December 13–December 23 and then entered a stable period. BF. 7 increased sharply around December 20–December 30 and then entered a stable period **(**Figure 5**)**.

## 4. Discussion

In this study, we reported the composition and dynamic trend of SARS-CoV-2 variants in Weihai from March 2022 to March 2023. We found that there have been significant changes in the variants and expansion patterns of SARS-CoV-2 before and after the adjustment of epidemic policies.

From March 2022 to November 2023, before the adjustment of the epidemic prevention policy, the COVID-19 cases in Weihai were mainly come from local clusters. During this period, one large-scale local clusters and three small-scale clusters occurred in Weihai. Through the analysis of phylogenetic tree, it can be found that:in the same local cluster, the sequences were highly homologous, and the whole genome sequences were almost identical. Combined with the results of epidemiological investigation, it can be found that these clusters were all caused by single source of infection cases contacting a large number of susceptible people in a short time. This confirmed the high transmissibility of the SARS CoV-2 variants (20, 21), and also showed the effectiveness of the COVID-zero strategy.In addition,we found that the variants that caused the BA.2 cluster, BA.1.1 cluster and BA.5.2.1 cluster were approximately the same as SARS-CoV-2 variants that circulating around the world during the same period.From this, it can be speculated that the three outbreaks may have been caused by imported cases. By comparing the sequences between the foreign imported cases and the local cases during the same period, it can be found that although the lineages of the SARS-CoV-2 variants were largely identical between foreign imported cases and local cases at the same time, but the sequences were not highly homologous, so these three local clusters may not be caused by these foreign imported cases.Instead, they were may be caused by domestic imported cases or foreign imported cases that we were not been monitored. We found 13 sequences of the gamma VOC (P.1.15) in June 2022 in Weihai. Since the gamma VOC(P.1.15) was only prevalent in Argentina and Chile from April to September 2021 (22). Yu (23) speculated that it was possible that the gamma variant endemic in South America in 2021 infected cold chain operation workers in Weihai through frozen squid contaminated by SARS-CoV-2. Studies have shown that frozen seafood cargoes contaminated by the SARS-CoV-2 can survive for a long time at low temperatures (24), since Weihai has a large number of foreign trade aquatic products companies handling cold chain seafood cargoes, this suggests that the local cluster may be caused by cold chain seafood cargoes contaminated by SARS-CoV-2.

In December 2022, With the adjustment of epidemic prevention policy in China, the genomic surveillance of SARS-CoV-2 variants was changed to once a week in Weihai (25). By analyzing the SARS-CoV-2 variants in Weihai from December 2022 to March 2023, we can find that the variants of BF.7 and BA.5.2 were dominant strains. The SARS-CoV-2 surveillance results from the China CDC showed that (26): from December 1, 2022 to April 13, 2023, the BA.5.2 accounted for 65.4% and BF.7 accounted for 32.8% in Chinese mainland. This indicated that dominant strains of Weihai were consistent with Chinese mainland during this period. The phylodynamic analysis showed that a large amount of genetic diversities of BF.7 and BA.5.2 appeared in December, and the effective population size of BF.7 and BA.5.2 also increased explosively in December. This may be the combined result of the adjustment of epidemic policy in early December and the high fitness of these two variants. In addition, we found that the effective population size of BF.7 expanded later than BA.5.2. This may be due to the fact that BA.5.2 had sporadic cases in early November,suggesting that BA. 5.2 had cryptic transmission during this period, while BF.7 appeared at the end of November. Therefore, after the adjustment of the epidemic policy, the expansion of BA.5.2 was earlier than that of BF.7.It should be noted that within 3 months after the adjustment of the epidemic policy, except for 3 BN.1 cases, no other variants other than BA.5.2 and BF.7 were monitored in Weihai for the time being, and neither the internationally prevalent BQ.1 variant nor the XBB variant appeared (27, 28).It probably because the founder effect established by these two variants in the short term was still effective (29, 30). However, with the continued evolution of SARS-CoV-2 variants and increased population movement, variants with higher transmissibility or higher immune escape may replace the currently prevalent variants in the near future. Therefore, SARS-CoV-2 genomic surveillance remains of great importance.

This study has some limitations. After the adjustment of the epidemic policy in December, the laboratory was unable to obtain the exact number of COVID-19 cases, so there was some sampling bias in the selection of samples for sequencing.In addition,the COVID-19 surveillance for imported personnel has been greatly reduced after December,which will also lead to a certain degree of error in our statistics.

In conclusion, we reported the composition and dynamic trend of SARS-CoV-2 variants in Weihai before and after the adjustment of the epidemic policy from March 2022 to March 2023. The dominant variants in Weihai were the same as the SARS-CoV-2 variants circulating globally at the same time, and no persistently dominant variants or new lineages were identified. Although COVID-19 no longer constitutes a “public health emergency of international concern,” long-term genomic surveillance of SARS-CoV-2 is still important in the future due to the continuous evolution and rapid mutation of SARS-CoV-2.

## Data availability statement

The datasets presented in this study can be found in online repositories. The names of the repository/repositories and accession number(s) can be found in the article/Supplementary material.

## Ethics statement

The studies involving humans were approved by Shandong Center for Disease Control and Prevention. The studies were conducted in accordance with the local legislation and institutional requirements. The human samples used in this study were acquired from gifted from another research group. Written informed consent for participation was not required from the participants or the participants' legal guardians/next of kin in accordance with the national legislation and institutional requirements.

## Author contributions

XL: Formal analysis, Investigation, Methodology, Validation, Writing – original draft. YZ: Formal analysis, Methodology, Writing – review & editing. JZ: Project administration, Supervision, Writing – review & editing. ZS: Investigation, Resources, Writing – original draft. XQ: Investigation, Resources, Writing – original draft. MW: Investigation, Resources, Writing – original draft. TM: Investigation, Resources, Writing – original draft. JL: Data curation, Investigation, Resources, Writing – original draft.
